# Distinct Effects on Diversifying Selection by Two Mechanisms of Immunity against *Streptococcus pneumoniae*

**DOI:** 10.1371/journal.ppat.1002989

**Published:** 2012-11-08

**Authors:** Yuan Li, Todd Gierahn, Claudette M. Thompson, Krzysztof Trzciński, Christopher B. Ford, Nicholas Croucher, Paulo Gouveia, Jessica B. Flechtner, Richard Malley, Marc Lipsitch

**Affiliations:** 1 Department of Epidemiology and Department of Immunology & Infectious Diseases, Harvard School of Public Health, Boston, Massachusetts, United States of America; 2 Genocea Biosciences, Inc., Cambridge, Massachusetts, United States of America; 3 Department of Pediatric Immunology and Infectious Diseases, Wilhelmina Children's Hospital, University Medical Center Utrecht, Utrecht, The Netherlands; 4 Division of Infectious Diseases, Department of Medicine, Boston Children's Hospital and Harvard Medical School, Boston, Massachusetts, United States of America; Malawi-Liverpool-Wellcome Trust Clinical Research Programme, Malawi

## Abstract

Antigenic variation to evade host immunity has long been assumed to be a driving force of diversifying selection in pathogens. Colonization by *Streptococcus pneumoniae*, which is central to the organism's transmission and therefore evolution, is limited by two arms of the immune system: antibody- and T cell- mediated immunity. In particular, the effector activity of CD4^+^ T_H_17 cell mediated immunity has been shown to act *in trans*, clearing co-colonizing pneumococci that do not bear the relevant antigen. It is thus unclear whether T_H_17 cell immunity allows benefit of antigenic variation and contributes to diversifying selection. Here we show that antigen-specific CD4^+^ T_H_17 cell immunity almost equally reduces colonization by both an antigen-positive strain and a co-colonized, antigen-negative strain in a mouse model of pneumococcal carriage, thus potentially minimizing the advantage of escape from this type of immunity. Using a proteomic screening approach, we identified a list of candidate human CD4^+^ T_H_17 cell antigens. Using this list and a previously published list of pneumococcal Antibody antigens, we bioinformatically assessed the signals of diversifying selection among the identified antigens compared to non-antigens. We found that Antibody antigen genes were significantly more likely to be under diversifying selection than the T_H_17 cell antigen genes, which were indistinguishable from non-antigens. Within the Antibody antigens, epitopes recognized by human antibodies showed stronger evidence of diversifying selection. Taken together, the data suggest that T_H_17 cell-mediated immunity, one form of T cell immunity that is important to limit carriage of antigen-positive pneumococcus, favors little diversifying selection in the targeted antigen. The results could provide new insight into pneumococcal vaccine design.

## Author Summary

*Streptococcus pneumoniae*, or pneumococcus, is a leading cause of morbidity and mortality in young children and elderly persons worldwide. Current pneumococcus vaccines target a limited number of clinically important serotypes, while strains with serotypes not targeted by current vaccines are increasing in importance in both carriage and invasive disease. As a result, there has been a substantial interest to develop novel, cost-effective vaccines based on protein antigens from pneumococcus. To this end, it is critical to understand how the human immune system exerts selection pressures on the targeted antigens. Two immune mechanisms targeting pneumococcal protein antigens have been documented, mediated by antibody and T cells, respectively. In this study, we screened for pneumococcal antigens that are commonly recognized by human CD4^+^ T_H_17 cells. Using a mouse model of pneumococcal colonization, we demonstrate that T_H_17 cell-based immunity almost equally reduces colonization by both an antigen-positive strain and a co-colonizing, antigen-negative strain. Furthermore, we demonstrate that the DNA sequences of T_H_17 cell antigens demonstrate no detectable signs of being under selective pressure, unlike pneumococcal antigens known to be strong antibody targets. Thus, one form of the T cell-mediated immunity that is important to limit carriage of antigen-positive pneumococcus favors little diversifying selection in the targeted antigen. These results suggest evolution of escape from T_H_17 -based vaccines may be slower than from antibody-based vaccines.

## Introduction

Diversifying selection on genes encoding pathogen antigens is a well known effect of host immunity [1], [2]. Diversifying selection can maintain multiple alleles of a gene at appreciable frequencies in a population [3]. Acquired immune responses provide a fitness advantage for antigenic variants that evade immune recognition, reducing the probability that the allele encoding the targeted antigen will fix with a single allele. In viruses such as HIV [4], [5], [6] and influenza [7], [8], neutralizing antibody and cytotoxic T-lymphocytes (CTLs) drive antigenic diversification. Strong diversifying selection was also identified in major antigen genes in the malaria parasite *Plasmodium falciparum* [9], [10]. In bacteria, diversity of surface structures (such as capsular polysaccharides) that are targeted by host antibodies is thought to result from such diversifying selection [1]. However, a few exceptions exist. Measles virus antigens show little variation, partially because exposure to the virus would generate polyclonal antibodies that efficiently neutralize a broad range of antigenic variants [11]. Human T cell epitopes of *Mycobacterium tuberculosis* show a substantially lower level of sequence variation than seen in other genomic regions, suggesting T cell immune responses might limit diversification in the antigen genes [12]. Therefore, we hypothesized that the effect of host immunity on diversifying selection depends on the specific mechanism involved.

Recent studies have indicated that acquired immunity elicited by natural exposure to *Streptococcus pneumoniae* includes three distinct arms [13]: (1) type-specific, antibody-mediated immunity to the highly variable polysaccharide capsule [14], [15], [16], [17], (2) antibody-mediated immunity to pneumococcal proteins, some of which are variable and some of which are more conserved [15], [18], [19], [20], [21], [22], [23], [24], and (3) CD4^+^ T_H_17 cell- mediated, antibody independent immunity to pneumococcal proteins and to the cell-wall polysaccharide [15], [25], [26], [27], [28]. The first two forms of immunity are thought to operate by the standard mechanisms of antibody binding to surface antigens, leading to opsonophagocytosis, reduced attachment and/or other mechanisms of reduced colonization [22], [29]. In the last form of immunity, antigen-specific CD4^+^ T_H_17 cells secrete interleukin (IL)-17A, leading to the activation and recruitment of effector cells (neutrophils and macrophages) that then kill pneumococci [25], [30], [31], [32]. T_H_17 cell-mediated immunity primarily accelerates the clearance of pneumococcus rather than preventing initiation of carriage [31]. Even in combination, these forms of immunity to *S. pneumoniae* are imperfect. Humans can be repeatedly colonized despite the immune responses from multiple arms.

While antibody binding is by definition specific to bacteria bearing the target antigen, we have previously shown that the CD4^+^ T_H_17-based effector activity may extend beyond antigen-expressing bacteria, accelerating the clearance of co-colonized pneumococci that even do not bear the relevant antigen [23]. It is unclear whether CD4^+^ T_H_17-mediated immunity would still create a fitness advantage for antigenic variants and thus promote diversifying selection on the genes encoding the targets of such immunity in *S. pneumoniae*.

Here we report the assessment of two hypotheses: first, a competition assay was performed to examine whether an antigen-negative strain shows a colonization advantage over the antigen-positive strain in mice with antigen-specific T_H_17 immunity. Second, pneumococcal genes that show signs of being under diversifying selection were systematically identified and their association with either Antibody antigens or T_H_17 antigens was examined. The results indicate little evidence of diversifying selection in the targets of CD4^+^ T_H_17 cell immunity, unlike the targets of antibody immunity.

## Results

### CD4^+^ T_H_17 cell-mediated immunity to pneumococcal carriage provides only weak selection for antigenic variation

Immunization with a pneumococcal whole cell vaccine displaying a peptide from ovalbumin (OVA^323–339^) delivered with cholera toxin (CT) adjuvant results in CD4^+^ T_H_17 cell-mediated and antibody-independent protection against subsequent pneumococcal colonization [23]. To examine whether the T_H_17 cell immunity against *S. pneumoniae*, given its *in trans* clearance effect [23], allows a competitive advantage for a non-recognizable (antigen-negative) strain, twenty BALB/c mice were immunized by either ovalbumin with adjuvant (OVA+CT) or adjuvant alone (CT). The mice were challenged with a 1∶1 mix of an antigen-negative strain (AVO) and an antigen-positive strain (OVA). The two strains were isogenic except that only the OVA strain displays OVA^323–339^ peptides that can be recognized by the ovalbumin-induced, T_H_17 immunity in mice [23]. The AVO strain can be viewed as an antigenic variant of the OVA strain and the AVO/OVA ratio would increase if there were a competitive advantage for the antigen-negative strain.

**Figure 1:**
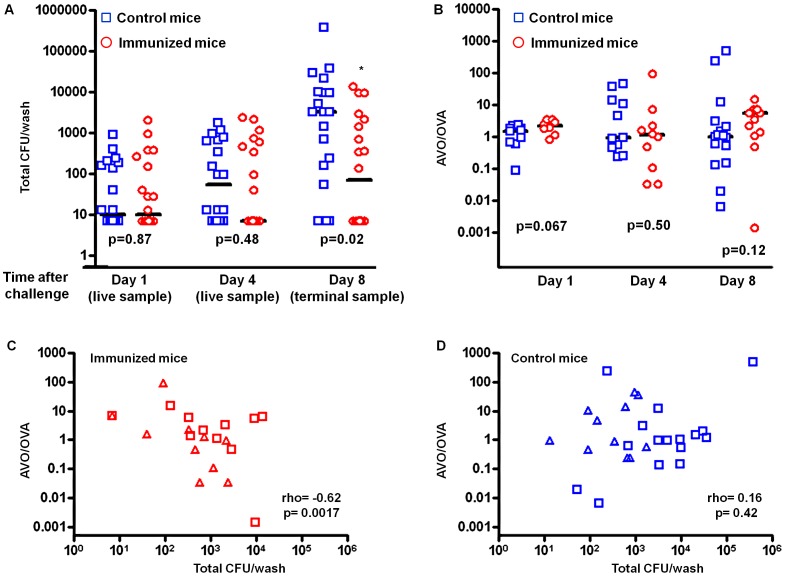
The benefit of antigenic variation in CD4+ T_H_17 epitope is limited. BALB/c mice were immunized by either CT alone (CT) or CT and ovalbumin (CT+OVA). All mice were challenged with a 1∶1 mixture of the antigen-negative (AVO) strain and the antigen-positive (OVA) strain. The density of intranasal colonization by pneumococcus in each mouse was determined on days 1, 4, and 8 after challenge as described in the Methods. Total CFU counts are shown in (A). The ratio between the two strains in each mouse was determined (B). The p values were derived from Mann-Whitney tests comparing the immunized with the control group on days 1, 4, and 8. Solid lines indicate group medians. The correlation between total CFU and the AVO/OVA ratio is shown for the immunized mice (C) and the control mice (D) that remained colonized on days 4 (triangle) and 8 (diamond).

The mixture of pneumococci colonized the ovalbumin-immunized and control mice equally well on day 1. No significant difference in colonization density was observed (Figure 1A, p = 0.87, Mann-Whitney test). By day 4, the median colonization density in ovalbumin-immunized mice was about 7-fold lower than that in the control mice, although the difference was not statistically significant (Figure 1A, p = 0.48, Mann-Whitney test). By day 8, the median colonization density in the immunized mice was about 40-fold lower than that in the control mice and the difference was statistically significant (Figure 1A, p = 0.02, Mann-Whitney test). The effect was consistent with an accelerated clearance of colonization mediated by T_H_17 immunity [31].

The AVO/OVA ratio remained approximately 1∶1 in the control mice during the course of the experiment (Figure 1B). The medians of log_10_ (AVO/OVA) were 0.185 (n = 10), −0.028 (n = 11), and 0.011 (n = 16) on days 1, 4 and 8, respectively (Table 1), indicating that the AVO strain was competitively neutral in the absence of antigen-specific immunity. In the ovalbumin-immunized mice, the medians of log_10_ (AVO/OVA) were 0.334 (n = 8), 0.042 (n = 10) and 0.730 (n = 13) on days 1, 4 and 8, respectively (Table 1). The median log_10_ (AVO/OVA) was not significantly different between the control and the immunized group on days 1, 4 or 8 (Figure 1B, p = 0.067, p = 0.50, and p = 0.12, respectively, Mann-Whitney test), although there was a trend toward an increase in AVO/OVA ratio in the immunized mice.

**Table 1:** Analysis of competitive advantage for the antigen-negative strain.

Day	Group	Sample size	Median log_10_(AVO/OVA)	P-value^a^	95% Confidence Interval^b^
1	Control	n = 10	0.185	0.067	(−0.006, 0.563)
	Immunized	n = 8	0.334		
4	Control	n = 11	−0.028	0.50	(−1.437, 0.456)
	Immunized	n = 10	0.0420		
8	Control	n = 16	0.011	0.12	(−0.232, 1.015)
	Immunized	n = 13	0.730		

a Two-sided Mann-Whitney test of equal median log_10_ (AVO/OVA).

b Nonparametric confidence interval for median of the difference in log_10_(AVO/OVA).

To better quantify the potential competitive advantage for the antigen-negative strain, we constructed nonparametric confidence intervals for the median of the difference in log_10_ (AVO/OVA) between the immunized group and the control group (Table 1). A median greater than 0 would indicate a competitive advantage for the AVO strain in the immunized group. The 95% confidence intervals for median difference in log_10_ (AVO/OVA) were (−0.006, 0.563), (−1.437, 0.456), and (−0.2319, 1.015) on days 1, 4, and 8, respectively (Table 1). Thus, the loss of an antigen was unlikely to provide a more than 10.4-fold (1.015 log_10_) median increase in competitive advantage for the AVO strain by day 8. We also note that the increased frequency of AVO strains was almost entirely found in mice who have nearly cleared colonization (Figure 1C). In absolute CFU numbers, therefore, the relative advantage is unlikely to be associated with much overall superiority.

In mice that remain colonized on days 4 and 8, a negative correlation between the AVO/OVA ratio and total CFU recovered was observed in the immunized group (Figure 1C) but not in the control group (Figure 1D). These results suggested that the antigen-negative strain gains a relative advantage only for the period where bacterial numbers are rather low.

### Identification of human CD4^+^ T_H_17 antigens in pneumococcus

**Figure 2:**
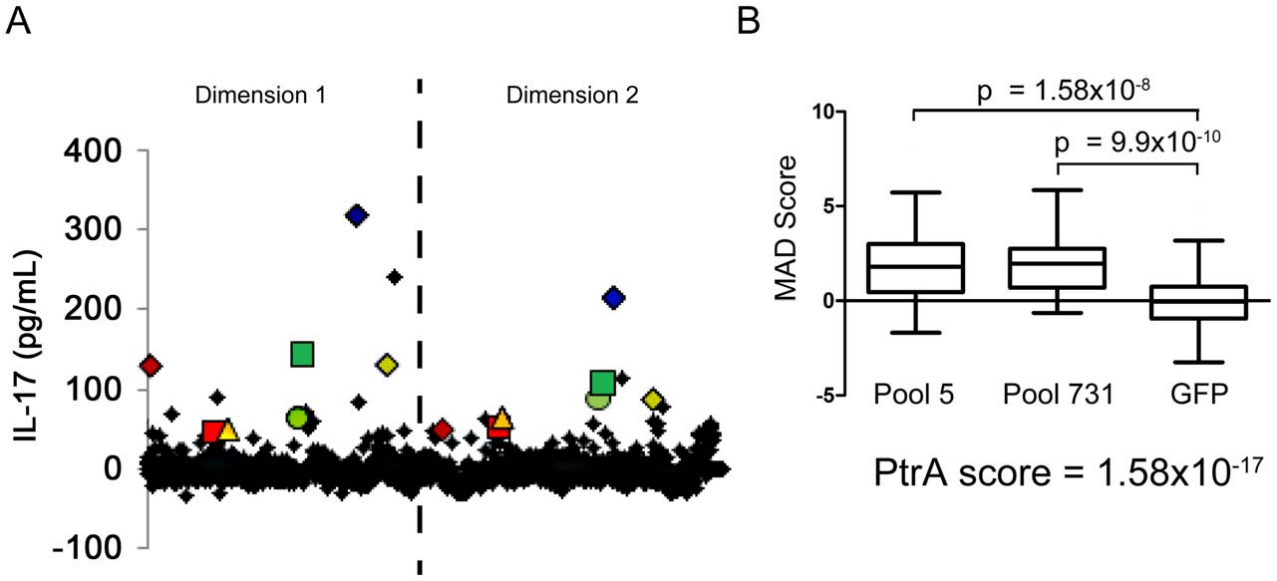
Identification of antigens recognized by human T_H_17 cells. (A) The average of duplicate ELISA measurements of the IL-17A concentration in the supernatant for each protein pool is displayed. The dashed line separates data points from the two dimensions of the pooled library (see SI for details). Each pair of colored geometric data points marks the data from pools that contain the same clone. (B) The MAD score for each pool measured in screens of enriched T_H_17 cells from 36 subjects was compared to the MAD score for wells containing MoDCs pulsed with *E. coli*-expressing GFP using a two-tailed Mann-Whitney test. The antigenicity score for PtrA (SP0641.1), calculated by multiplying the p-values resulting from the Mann-Whitney test of the two pools containing the clone, is also displayed.

To determine whether CD4^+^ T_H_17 cell-mediated immunity to *S. pneumoniae* affects antigenic variation in the context of human colonization and disease, *S. pneumoniae* antigens recognized by human T_H_17 cells were identified. CD4^+^ T_H_ 17 cells were enriched from peripheral blood cells and IL-17A secretion in response to pneumococcal protein pools was measured by ELISA (see Materials and Methods, Figure S1, and Figure S2). To identify the common antigens in the sample population of 36 healthy adults, a Mann-Whitney test was used to compare normalized values for each pool to the normalized values for *E. coli* expressing GFP. Each protein was then ranked by its antigenicity score, which was calculated by multiplying together the p-values resulting from the Mann-Whitney test for both pools containing the protein, lower antigenicity scores indicating more commonly recognized antigens. An N-terminal fragment of PtrA (SP0641.1) was the most strongly recognized antigen in the screen with an antigenicity score of 1.58×10^−17^ (Figure 2B). Clones with a score less than 0.05 were defined as the common antigens (Table 2).

**Table 2:** Identification of human T-cell antigens in pneumococcus.

Antigenicity Sore	Clone	Function
1.58E-17	**SP0641.1**	**Serine protease**
2.55E-10	**SP1839**	**ABC transporter; ATP-binding/permease protein**
6.52E-10	**SP1323**	**hypothetical protein**
2.74E-09	**SP1072**	**DNA primase (dnaG)**
8.63E-09	**SP1434**	**ABC transporter; ATP-binding/permease protein**
1.77E-08	**SP1780**	**oligoendopeptidase F; putative**
3.85E-08	**SP0264**	**prolyl-tRNA synthetase (proS)**
9.68E-08	**SP1553**	**ABC transporter; ATP-binding protein**
3.33E-07	**SP0979**	**oligoendopeptidase F (pepF)**
3.36E-07	**SP0445**	**acetolactate synthase; large subunit; biosynthetic type (ilvB)**
3.48E-07	**SP2136**	**choline binding protein PcpA (pcpA)**
6.00E-07	**SP2032**	**transcriptional regulator; BglG family**
9.37E-07	**SP1739**	**KH domain protein**
1.68E-06	**SP2216**	**secreted 45 kd protein (usp45)**
2.32E-06	**SP0475**	**hypothetical protein**
2.92E-06	**SP1976**	**pyruvate formate-lyase-activating enzyme (pflA)**
4.92E-06	**SP0266**	**glucosamine–fructose-6-phosphate aminotransferase; isomerizing**
6.37E-06	**SP0139**	**conserved domain protein**
8.35E-06	**SP1310**	**IS1381; transposase OrfA**
1.70E-05	**SP1662**	**ylmH protein (ylmH)**
1.79E-05	**SP1251**	**endonuclease; putative**
2.03E-05	**SP2029**	**preprotein translocase; YajC subunit (yajC-2)**
4.53E-05	**SP0148**	**ABC transporter; substrate-binding protein**
4.98E-05	**SP1730**	**hypothetical protein**
8.75E-05	**SP0040**	**hypothetical protein**
1.07E-04	**SP1305**	**hypothetical protein**
1.10E-04	**SP0777**	**hypothetical protein**
1.26E-04	**SP0909**	**conserved hypothetical protein**
1.37E-04	**SP0017**	**IS1167; transposase; degenerate**
1.69E-04	**SP1717**	**ABC transporter; ATP-binding protein**
2.03E-04	**SP1476**	**hypothetical protein**
2.04E-04	**SP1594**	**IS3-Spn1; hypothetical protein; interruption**
2.21E-04	**SP1774**	**transcriptional regulator; putative**
2.85E-04	**SP0471**	**conserved hypothetical protein**
2.99E-04	**SP1465**	**hypothetical protein**
3.01E-04	**SP0955**	**competence protein CelB (celB)**
3.21E-04	**SP0310**	**PTS system; IIC component**
3.23E-04	**SP0394.2**	**PTS system, mannitol-specific IIBC components**
3.37E-04	**SP1254**	**hypothetical protein**
3.42E-04	**SP0723**	**conserved domain protein**
3.57E-04	**SP1278**	**pyrimidine operon regulatory protein (pyrR)**
3.76E-04	**SP1484**	**transposase family protein; authentic frameshift**
4.39E-04	**SP0871**	**conserved hypothetical protein**
6.77E-04	**SP1740**	**conserved hypothetical protein**
8.33E-04	**SP0605**	**fructose-bisphosphate aldolase (fba)**
8.99E-04	**SP1684**	**PTS system; IIBC components**
9.15E-04	**SP1912**	**hypothetical protein**
1.09E-03	**SP0698.1**	**hypothetical protein**
1.12E-03	**SP0575**	**helicase; putative**
1.33E-03	**SP0257**	**group II intron; maturase; degenerate**
1.49E-03	**SP0384.1**	**FMN-dependent dehydrogenase family protein**
1.54E-03	**SP0987**	**hypothetical protein**
1.55E-03	**SP1431**	**type II DNA modification methyltransferase; putative**
1.89E-03	**SP0809**	**hypothetical protein**
2.44E-03	**SP1314**	**IS66 family element; Orf1**
4.19E-02	**SP1385**	**hypothetical protein**
4.86E-02	**SP1384**	**conserved hypothetical protein**
4.87E-02	**SP0279**	**conserved hypothetical protein**

### Detection of diversifying selection in pneumococcus

To evaluate genetic diversity and the underlying selection pressure on pneumococcal proteins, we systematically examined protein-encoding regions from the genome sequence data of 39 publicly-available pneumococcus strains for evidence of diversifying selection. Based on information accompanying the genome sequence data, the collection of strains covered 14 common serotypes (Table S1 in Text S1). Although the strains used in our study are not a random sample of any population and may overrepresent clinical (invasive) isolates, the distribution of serotype frequency in this study was reasonably consistent with distribution reported in human carriage [33] (Figure S3).

**Figure 3:**
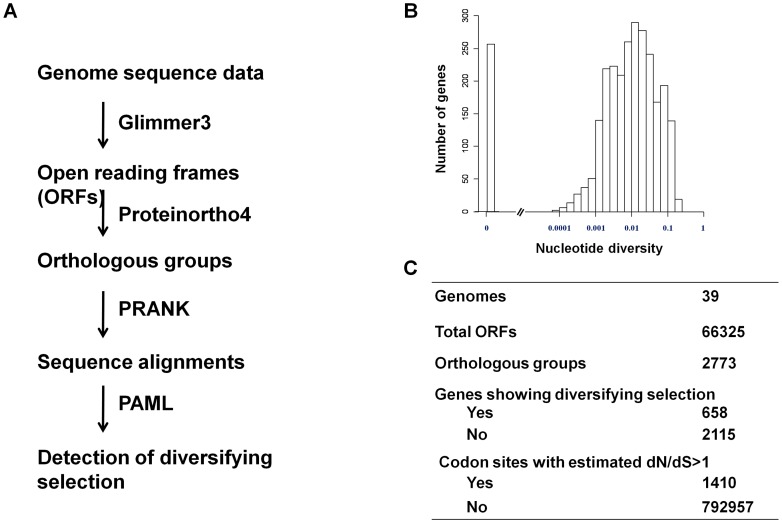
Detection of diversifying selection in pneumococcus. (A) Schematic of the workflow showing the procedures and software used to detect of diversifying selection in pneumococcus. (B) The distribution of nucleotide diversity (π) among pneumococcal genes. (C) A summary of number of genes and codon sites that show sign of being under positive selection.

A flowchart of the analysis is shown in Figure 3A. Open reading frames (ORFs) that were inferred to represent the same gene in different strains were grouped together to form an orthologous group. A total of 2773 unique unambiguous groups were generated by the Proteinortho4 software [34]. Sequence alignment of genes within an orthologous group was performed using the PRANK software [35]. Extensive sequence variation was observed for many pneumococcal protein-encoding genes. The nucleotide diversity for a gene ranged from 0 to 0.23 with a median of 0.0091 (Figure 3B).

To identify pneumococcal genes that show signs of being under diversifying selection, we analyzed the non-synonymous to synonymous substitution (dN/dS) ratio for codon sites in each gene using the PAML package as described by Yang [36]. Signs of being under diversifying selection were detected by a likelihood ratio test in which a null model (dN/dS < = 1 for all codons) was compared with an alternative model (dN/dS>1for at least one codon), as described in the Materials and Methods. We concluded signs of diversifying selection for a gene if the null model was rejected at the significance level of 0.05. By this criterion, 658 genes (23.7%) showed signs of being under diversifying selection. The subsequent Bayes Empirical Bayes (BEB) analysis [37] identified 1410 codon sites, or 0.178% of total codon sites, to be under diversifying selection (Figure 3C). Codon sites under diversifying selection were enriched in cell envelope genes (Table S2 in Text S1), consistent with that interaction with antibodies might be a source of selection pressure on the pneumococcal protein antigens.

### A link between immune recognition and diversifying selection

We hypothesized that if human immunity had promoted diversifying selection in pneumococcal antigens, the antigen genes would exhibit higher sequence diversity than non-antigen genes. Genes encoding CD4^+^ T_H_ 17 antigens were identified as described above. Genes encoding Antibody antigens were obtained from the list published by Giefing *et al* [24]. TIGR4 genes belonging to an orthologous group of two or more genes were analyzed, including 1648 non-antigens, 48 T_H_17 antigens and 80 Antibody antigens. In addition, the regions of Antibody antigens genes that included epitopes were also noted by Giefing *et al*., facilitating our comparisons of non-antigens, Antibody antigen-encoding genes, and the epitope-containing and non-epitope-containing regions of these antigen-encoding genes.

**Figure 4:**
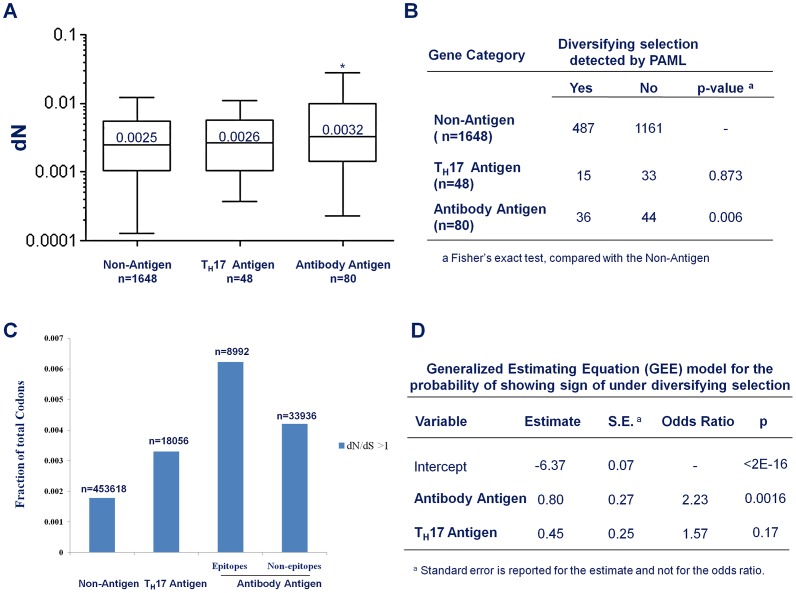
Antibody recognition is associated with stronger diversifying selection. (A) Box plot to compare the average non-synonymous substitution rate (dN) of non-antigens, the Antibody antigens and CD4^+^ T_H_17 antigens. * p<0.05, Mann-Whitney test, compared with non-antigens (B) The fraction of genes that show evidence of being under diversifying selection in non-antigens, Antibody antigens and CD4^+^ T_H_17 antigens. (C) The fraction of codons with dN/dS>1 in non-antigen, the T_H_17 antigens, and the epitope and the non-epitope regions of the Antibody antigens. (D) Output of a generalized-estimating-equation (GEE) analysis for the effect of antibody-recognition (Antibody Antigen) and CD4^+^ T_H_17 cell- recognition (T_H_17 Antigen) on the probability that a gene shows signs of being under diversifying selection.

The average non-synonymous substitution rate (dN) of Antibody antigens was significantly higher than that of non-antigens (Figure 4A; median 0.0032 vs. 0.0025; p = 0.022, Mann-Whitney test). However, there was no significant difference in dN between T_H_17 antigens and non-antigens. (Figure 4A; median 0.0026 vs. 0.0025; p = 0.65, Mann-Whitney test). Genes encoding Antibody antigens also showed a significantly higher proportion of genes with signs of being under diversifying selection (Figure 4B, OR = 1.95, p = 0.006, Fisher's Exact test). In contrast, T_H_17 antigen genes showed no evidence of being under diversifying selection (Figure 4B, OR = 0.77; p = 0.52; Fisher's Exact test).

Not all codon sites within a gene need be under the same selective force. To understand the contribution of host immunity to diversifying selection, we were particularly interested in whether the codon sites that did show an estimated dN/dS ratio greater than 1 were equally distributed among antigen categories. We found that 0.183% of the codon sites located in the non-antigen genes showed dN/dS ratio greater than 1 (Figure 4C). For codon sites in the CD4^+^ T_H_ 17 antigen genes, a higher fraction (0.33%, Figure 4C) showed a dN/dS ratio greater than 1. An even higher fraction (0.46%, Figure 4C) of the Antibody antigen codon sites showed a dN/dS ratio greater than 1. Furthermore, within the Antibody antigens, the regions in antibody epitopes showed a higher density of codon sites with dN/dS greater than 1 than the non-epitope regions (0.62% vs. 0.42%, Figure 4C). Thus, the genomic regions that interact with antibody-mediated immunity appeared to be more enriched for codon sites with signs of being under diversifying selection, with a weaker signal of diversifying selection in the CD4^+^ T_H_17 antigens.

To account for correlations between different codon sites within a gene and for differences in gene length that would make longer genes more likely, by chance alone, to have sites with elevated dN/dS ratios, we employed a generalized-estimating-equation (GEE) model to examine the “population-averaged” effect of being recognized by human immunity on the probability that a gene is under diversifying selection [38]. Essentially, we treated the status of each individual codon in a gene (whether or not the codon showed sign of being under diversifying selection) as the outcome of a repeated measurement for the status of the gene (whether or not the gene showed sign of being under diversifying selection). During model fitting, the covariance structure across codon sites within a gene was treated as a nuisance parameter. The output of the model fitting showed that being an Antibody antigen is a highly significant predictor for being under diversifying selection (Figure 4D; OR = 2.23, p = 0.0016) and being a T_H_17 antigen is a weaker, and not statistically significant predictor (Fig. 4D, OR = 1.57, p = 0.17). Taken together, these results indicated that antibody immunity made a greater contribution than CD4^+^ T_H_ 17 cell immunity to diversifying selection on antigen genes in *S. pneumoniae*.

To examine the robustness of our results, we carried out the analysis of diversifying selection using a different alignment algorithm [39], as well as another evolution model proposed by Wilson *et al*., which allows estimation of the dN/dS ratio in the presence of recombination [40]. All analyses yielded qualitatively similar results (Table S3 and Table S4 in Text S1).

## Discussion

In this study, we investigated the contribution of host immunity to the diversifying selection in *S. pneumoniae*. We found that CD4^+^ T_H_17 cell-mediated immunity, elicited by exposure to pneumococci bearing a targeted antigen, cleared pneumococci that do not bear this antigen *in trans* almost as efficiently as it cleared the antigen-bearing cells. Thus, T_H_17 cell immunity limited the competitive benefit of antigenic variation within a colonized host, potentially reducing a driving force of diversifying selection. Consistent with this notion, we found a weak, and not statistically significant association between diversifying selection and recognition by human T_H_17 cell immunity. We hypothesize that this lack of selection is due to *in trans* killing of antigen-negative bacteria by innate cells recruited through T_H_17 cells recognition of antigen-expressing bacteria. However, the promiscuity of CD4^+^ T cell epitope recognition [41] could also play a role as it may be more difficult for bacteria to mutate the recognized antigens to avoid T cell recognition. In contrast to T_H_17 antigens, there was a significant association between recognition by human antibody and diversifying selection on the antigen. These data suggest that these two mechanisms of acquired immunity exert distinct selection forces on their respective antigens in *S. pneumoniae.*

We observed that an antigen-negative (AVO)/antigen-positive (OVA) ratio higher than 1 was associated with lower CFU in the ovalbumin-immunized mice but not in the control mice. This supported the antigen-specificity of the immunity recalled by the OVA strain. In principle, there are three stages of the pneumococcal life cycle in which escape from immunity might be beneficial: (1) an advantage for an escape variant by mutation or deletion of an antigen that is the target of an immune response during infection; (2) an advantage for a variant in colonizing a host already responding to a “wild-type” strain that is resident and targeted by the host's response; (3) an advantage for a variant in colonizing a host that is currently uncolonized with any pneumococcal strain, but has immunity to wild-type alleles of the antigen from previous exposure. *Cis*-acting immune effectors, such as antibodies, would be expected to provide an advantage for a variant at all three of these stages. Our animal experiments suggest that for CD4^+^ T_H_17 cells, the advantage of an immune-escape variant would be small at stages 1 and 2, because of *in-trans* killing; the first stage is particularly important because this is where a variant would likely first arise. Still, one would expect some advantage for CD4^+^ T_H_17 cell escape variants at the third stage – colonization of an uncolonized but partially immune host; this possibly may account for the weaker, less statistically convincing evidence of enrichment for diversifying selection in CD4^+^ T_H_17 antigens.

Escape from CD4^+^ T_H_ 17 cell immunity in our in vivo model should be more favored than in natural settings, for two reasons. First, we constructed a model in which the T_H_17 epitope was completely deleted (and replaced with the reverse amino acid sequence), rather than creating a point mutation; given the promiscuity of T cell responses, many point mutations might make little or no difference to T cell recognition. Second, natural exposure to pneumococci would induce immunity to multiple T cell (and antibody) antigens, so that escape from a single response would not necessarily create a major advantage. The fact that we saw a modest benefit of losing the sole CD4+ T cell epitope against which the mice had been immunized argues that the benefit would be even weaker under natural conditions.

The high throughput screen was designed to pick up the antigens with the strongest T_H_17 responses in the studied sample. This strength includes both frequency of response in the studied population and the strength of the response within individuals. The Mann-Whitney analysis does not allow us to define whether an antigen was positive in any given subject. However, if we use a different analysis method of taking antigens that induce a response greater than 1.2 MAD above the median, we find that the most common antigen was recognized by 47% of the subjects, with most antigens present in 10–20% of subjects (data not shown), indicating a reasonably broad T_H_17 response. We acknowledge that there are weaker responses in these individuals that may have not been detected, but we posit that any selective pressure on T_H_17 antigens should be more robust in the strongly recognized antigens. Since no association between signs of diversifying selection and the human T_H_17 antigens we identified was found, the observation supports our hypothesis that CD4^+^ T_H_17 cell immunity in humans allows minimal competitive benefit for antigenic variation in *S. pneumoniae*. It is also important to note that only antigens recognized by IL-17A secreting T cells were identified. If the antigens recognized by different T cell lineages are distinct [42], [43], other T cells lineages may exert stronger selective pressure depending on their mechanism of action.

We found that genomic regions that showed signs of being under diversifying selection were enriched in the antibody antigen genes and further enriched in the epitopes targeted by antibodies. This finding was consistent with the conventional understanding that avoidance of antibody-recognition can provide a substantial competitive benefit. The magnitude of the enrichment was consistently modest among all analyses. It is possible that multiple ways to avoid antibody-recognition exist, reducing the dependence on non-synonymous substitutions in the antigens. For example, antigens can be temporarily down regulated at the expression level to escape from host immunity, as was seen in the malaria parasite *Plasmodium falciparum* [44] and suggested for meningococci under vaccine pressure [45]. Antigens are also proteins carrying out physiological functions for the pathogen at the same time. They might be subjected to diversifying, purifying or other selective forces in addition to those imposed by acquired immunity. However, the significant association between antibody-recognition and diversifying selection despite these putative competing mechanisms suggested that antibodies impose a strong fitness cost on the antigen-bearing pneumococcus. In addition, it would be interesting to understand whether the diversifying selection differs in selected genes according to the invasive potential and transformability of the strain. Appropriate comparison would require much larger samples, which we hope to investigate in future studies.

CD4+ T subsets other than the T_H_17 cells, such as the IFN-γ producing T_H_1 cells, have been proposed to play important roles in the control of pneumococcal invasive disease [42], [43] but not, to our knowledge, colonization. In fact, in our colonization model, the IFN-γ mediated mechanism appeared to be dispensable [31]. Our screen would not have picked up antigens that elicited CD4+ T responses unless they also stimulated IL-17A production. Further work might address the contribution of other forms of T cell mediated immunity to diversifying selection.

This study suggests that CD4^+^ T_H_17 cell immunity creates little selective pressure for antigenic variation while efficiently protecting against pneumococcal colonization, and suggest that the reason for this lack of selection may be due to efficient *in trans* killing of antigenic variants arising within a host. It is conceivable that a vaccine designed to induce T_H_17 cell immunity might limit the immune escape of antigenic variants and result in broader and longer protection. To this end, further research is ongoing to characterize the major T_H_17 cell antigens in pneumococcus and identify methods for eliciting this type of immunity through vaccination [27], [46].

## Materials and Methods

### Ethics statement

All human subjects enrolled in this study provided written informed consent. The protocols for this study were IRB-approved by Quorum Review, Inc.

All animal work has been conducted in compliance with the Animal Welfare Act and the guidelines of the U.S. Public Health Service Policy on Humane Care and Use of Laboratory Animals, and specifically approved by the Institutional Animal Care and Use Committee (IACUC) of Harvard Medical School. (Animal Welfare Assurance of Compliance A3431-01 and AAALAC Accreditation #000009, 6/19/09)

### Strains and animals

The antigen-positive *S. pneumoniae* stain (OVA) was a serotype 6B strain 603 derivative that expressed the OVA^323–339^ peptide (ISQAVHAAHAEINEAGR) on the bacterial surface as fusion proteins with both pneumococcal surface protein A (PspA) and pneumolysin (Ply) [23]. To construct the antigen-negative *S. pneumoniae* (AVO), the OVA coding sequence in the *pspA* and *ply* loci of the OVA strain was replaced by a nucleotide sequence encoding the OVA^323–339^ peptide in reversed sequence (RGAENIEAHAAHVAQSI) by using a Janus-cassette mediated transformation protocol [47].

Wild-type, female BALB/c mice were obtained from the Jackson ImmunoResearch Laboratories, Barr Harbor, ME. All mice were 5 to 6 weeks old at the start of experiments and kept in a BL2 facility.

### Immunization and challenge

Ovalbumin (Sigma-Aldrich, St. Louis, MO) and cholera toxin (CT) mucosal adjuvant (List Biological Laboratories, Compel, CA) were purchased and stored according to the manufacturer's protocols. Mice were intranasally immunized twice, one week apart, with10 µL of PBS containing 10 µg Ovalbumin plus 1 µg CT (OVA+CT) or 1 µg CT alone (CT).

Four weeks after the second immunization, mice were inoculated intranasally with a mix of the OVA and the AVO strains in 10 µl of PBS containing approximately 5×10^6^ CFU of each strain. On days 1 and 4 after challenge, samples from live animals were collected by applying 10 µl of ice cold PBS to either nostril of a mouse and collecting droplets discharged by the animal. On day 8 after challenge, upper respiratory tract samples were collected post mortem from retrotracheal washes of sacrificed mice. Aliquots of sample were titered to determine the colonization density. The remaining samples were cultured on gentamicin plates overnight and the resulting colonies were harvested for genomic DNA extraction.

### Quantitative PCR

Genomic DNA was purified from cultures of samples collected from animals using DNeasy Blood and Tissue kit (QIAGEN, Valencia, CA). The OVA strain- and the AVO strain-specific primer sets were designed based on the nucleotide sequence difference in the *pspA* locus between the two strains. The quantity of strain-specific genomic DNA in a sample was determined by absolute quantification protocol. A standard curve was built for each qPCR plate and was based on two replicates. All samples were measured based on averaged value of qPCR duplicate. The CFU ratio between the two strains was calculated by using the absolute amount of OVA DNA and AVO DNA in the same sample. The detection limit of AVO/OVA ratio was set as from (1×total CFU)^−1^ to (1×total CFU). The qPCR-derived ratios outside this range were rounded to the nearest detection limit.

### Human CD4^+^ T_H_ 17 antigen screen

Approval for blood collection was obtained from the Institutional Review Boards of each institution. IL-17A-secreting CD4^+^ T cells were first enriched from peripheral blood cells using negative magnetic selection of CD4^+^ T cells and a previously published IL-17A cytokine capture protocol [48]. *S. pneumoniae-*specific T_H_17 cells were further enriched by culturing the cells with autologous monocyte-derived dendritic cells (MoDCs) pulsed with inactivated *S. pneumoniae.* IL-17A secretion from the cells was measured after three days of co-culture with MoDCs pulsed with *E. coli* expressing a previously validated 2,547 clone ORFeome library of the *S. pneumoniae* TIGR4 genome [49] arrayed in pools of four clones. Enriched cells from 36 peripheral blood samples were screened with the pooled library (see SI for methods detail). The results of the IL-17A ELISA were first normalized by plate by averaging the duplicates for each well, subtracting the plate median from each average and then dividing the result by the median absolute deviation of the plate, yielding the MAD score for each well in the screen. The most common antigens recognized by the population were identified by comparing the population response to each pool in the library to the measured responses to the all the wells that received *E. coli* expressing GFP using a one-tailed Mann-Whitney test. Each individual antigen was then scored by multiplying the p-values from the Mann-Whitney test of the two wells in which it was present.

### Genome sequences

Genome sequence data of 39 pneumococcal strains were retrieved from the NCBI FTP site, ftp://ftp.ncbi.nih.gov/genomes. The collection included 14 annotated genomes and 25 draft genomes. Accession numbers of genome sequence were listed in Table S1 in Text S1. For the annotated genomes, the annotation and nucleotide sequence of each gene were downloaded from the NCBI FTP site. For the draft genomes, putative protein-encoding genes were identified by using the Glimmer3 software [50]. Orthology analysis of pneumococcal proteins was carried out by using the Proteinortho4 software [34], which assigned orthologous proteins from different strains into a same orthologous group based on the reciprocal best alignment heuristic. Cellular roles of TIGR4 genes were categorized according to the JCVI Annotation Gene Attributes (http://cmr.jcvi.org).

### Analysis of the non-synonymous to synonymous rate ratio (dN/dS ratio)

The gene sequences of each orthologous group were aligned based on the amino acid sequences they encode (codon alignment) and a gene tree was constructed using either the ClustalW software or the PRANK software [35], [39]. A likelihood ratio test was applied to compare a null model with an alternative model of the distribution of the dN/dS ratio parameter, ω, among codon sites, as described in [37]. In the null model (nearly-neutral model), each codon site within a gene is assumed to be either under purifying selection (ω0<1) or under neutral evolution (ω1 = 1). In the alternative model (positive selection model), a codon site can be under purifying selection (ω0<1), under neutral evolution (ω1 = 1) or under diversifying selection (ω2>1). For each model, the log likelihood value was calculated by the CodeML program from the package PAML [36]. If the null model was rejected by the likelihood ratio test at a significance level of 0.05, the gene represented by the orthologous group would be considered as being under diversifying selection. For such genes, a Bayes Empirical Bayes (BEB) analysis implemented in the CodeML program [36] was used to determine the particular codon sites that were under diversifying selection.

The output file of the CodeML program included non-synonymous substitution rate (dN) derived from pair wise sequence comparison. The average dN for each orthologous group was estimated by averaging over all pair wise dNs.

The dN/dS ratio for codon sites was also estimated by a method developed by Wilson *et al*., which applied a population genetics approximation to the coalescent to accommodate recombination events [40]. The codon alignment of each orthologous group was analyzed by Omegamap software with a prior exponential distribution of ω and a prior ω mean of 1. Each codon site was assumed to have independent ω and the posterior distributions of ω were obtains by 500,000 iterations. A codon site was defined to show evidence of being under diversifying selection if 95% of its posterior distribution of ω was above 1. A gene was considered to show evidence of being under diversifying selection if any codon site within the gene showed sign of being under diversifying selection. The analyses took 3–4 weeks on a Linux cluster comprised of 4708 processor cores.

Statistical analysis was performed by using the R package (http://www.r-project.org). Graphs were created in Graphpad Prism and in Microsoft Excel.

List of NCBI-Gene ID numbers for genes and proteins mentioned in the text: 929896 (PspA), 931915 (Pneumolysin), 930590 (PtrA).

## Supporting Information

Figure S1 Enrichment of *S. pneumoniae*-specific T_H_17 cells. (A) CD4^+^ T cells purified from PBMCs by magnetic sorting were further enriched for IL-17A secreting cells through IL-17A capture and sorting. A portion of the enriched cells and unsorted CD4^+^ T cell population were nonspecifically expanded with α-CD3/α-CD28 antibody-coated beads for 12 days in the presence of IL-2 and then activated with PMA/ionomycin in duplicate wells. The average IL-17A concentration in the supernatant was measured by ELISA after three days of incubation and is plotted for each T cell population. (B) A portion of the two T cell populations nonspecifically expanded in part (a) were added to MoDCs that had been pulsed for one hour with inactivated *S. pneumoniae*. After 12 days, both the nonspecifically activated and *S. pneumoniae*-pulsed MoDC-activated T cells were added to fresh MoDCs that had been pulsed for two hours with either *S. pneumoniae* or media alone and then fixed with paraformaldehyde prior to addition of the T cells. The IL-17A concentration in the supernatant after three days of incubation was measured by ELISA and is displayed for each T cell population. US = unsorted, T_H_17 = enriched for T_H_17 cells, NS = nonspecifically activated for expansion, WCV = activated with *S. pneumoniae*-pulsed MoDCs for expansion. Error bars = 1 SD.
(TIF)

Figure S2 Pooling strategy for the clonal library. Each set of four consecutive plates in the clonal library were pooled with two different methods to create a two-dimensional library. The first dimension was created by pooling the same well in the four consecutive plates. The second dimension was created by pooling four consecutive rows on the same plate. The individual clone responsible for inducing a T cell response to a pool was identified by examining the four pools in the second dimension that contain one of the clones present in the stimulating pool in the first dimension. The clone that is present in a positive pool in both dimensions of library is designated the stimulating clone.
(TIF)

Figure S3 Serotype distribution of strains analyzed in this study is compared with what was reported for human carriage by Bogaert *et al* [33]. The Spearman's rank correlation coefficient (rho) is shown.
(TIF)

Text S1 The file includes supplementary methods, supplementary figure legends, table S1: genomic sequence data used in this study, table S2: distribution of codon sites under diversifying selection, table S3: effects of sequence alignment and evolution model on the detection of diversifying selection, and table S4: effects of sequence alignment and evolution model on the GEE analysis.
(DOCX)
