# Supplementary figures and images for: Distinct Effects on Diversifying Selection by Two Mechanisms of Immunity against Streptococcus pneumoniae
Source: PLoS Pathog. 2012 Nov 8;8(11):e1002989. doi: 10.1371/journal.ppat.1002989 (PMC3493470; doi:10.1371/journal.ppat.1002989)

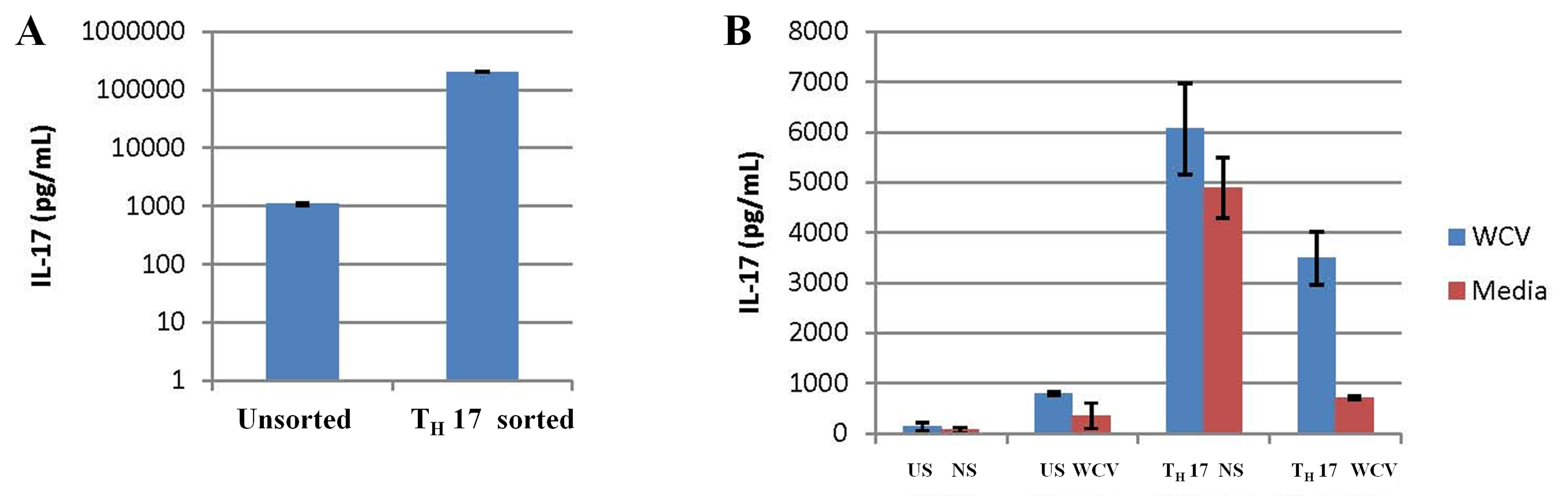

Supplement: Figure S1 — Enrichment of S. pneumoniae-specific TH17 cells. (A) CD4+ T cells purified from PBMCs by magnetic sorting were further enriched for IL-17A secreting cells through IL-17A capture and sorting. A portion of the enriched cells and unsorted CD4+ T cell population were nonspecifically expanded with α-CD3/α-CD28 antibody-coated beads for 12 days in the presence of IL-2 and then activated with PMA/ionomycin in duplicate wells. The average IL-17A concentration in the supernatant was measured by ELISA after three days of incubation and is plotted for each T cell population. (B) A portion of the two T cell populations nonspecifically expanded in part (a) were added to MoDCs that had been pulsed for one hour with inactivated S. pneumoniae. After 12 days, both the nonspecifically activated and S. pneumoniae-pulsed MoDC-activated T cells were added to fresh MoDCs that had been pulsed for two hours with either S. pneumoniae or media alone and then fixed with paraformaldehyde prior to addition of the T cells. The IL-17A concentration in the supernatant after three days of incubation was measured by ELISA and is displayed for each T cell population. US = unsorted, TH17 = enriched for TH17 cells, NS = nonspecifically activated for expansion, WCV = activated with S. pneumoniae-pulsed MoDCs for expansion. Error bars = 1 SD. (TIF) [file ppat.1002989.s001.tif]

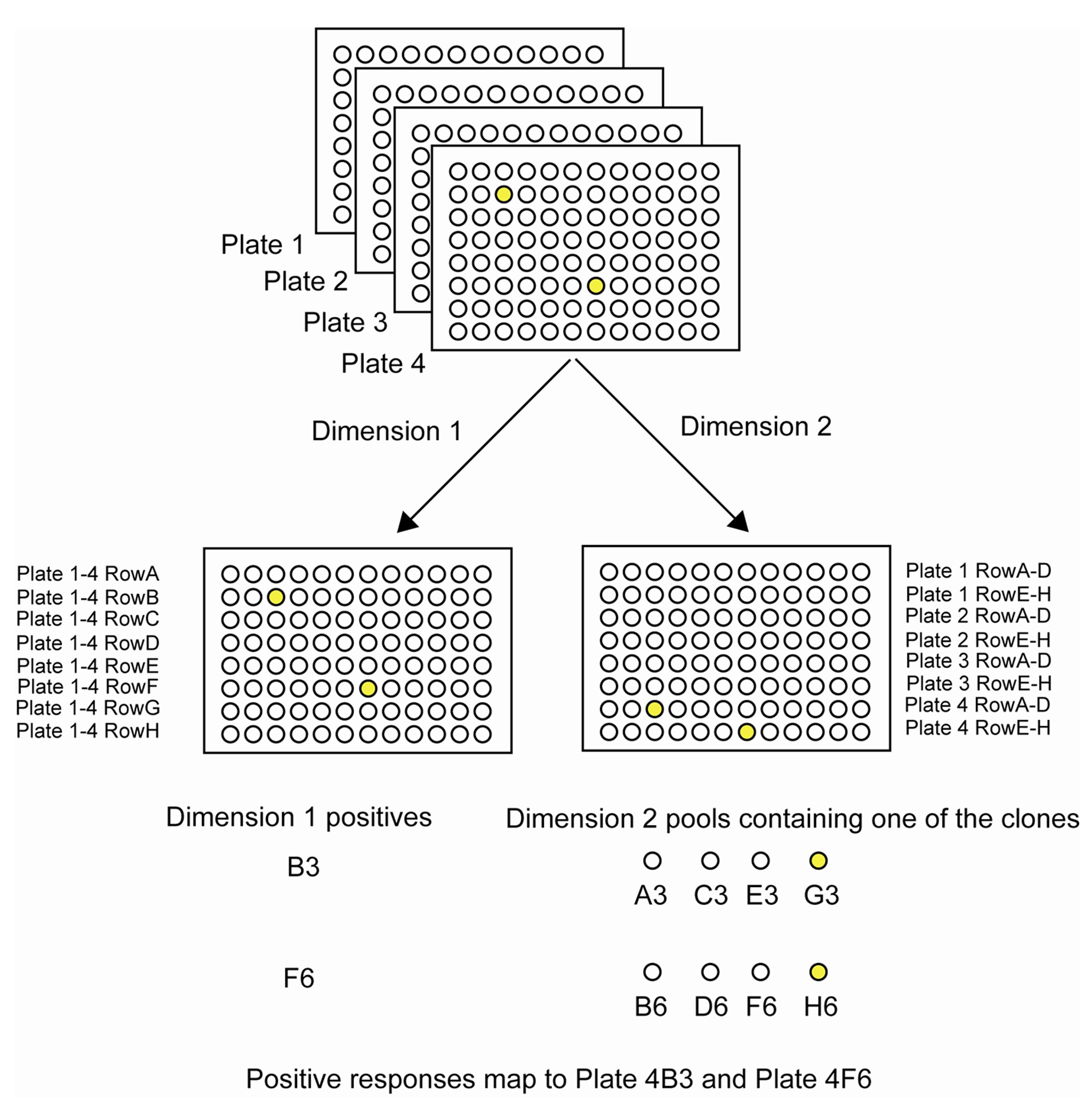

Supplement: Figure S2 — Pooling strategy for the clonal library. Each set of four consecutive plates in the clonal library were pooled with two different methods to create a two-dimensional library. The first dimension was created by pooling the same well in the four consecutive plates. The second dimension was created by pooling four consecutive rows on the same plate. The individual clone responsible for inducing a T cell response to a pool was identified by examining the four pools in the second dimension that contain one of the clones present in the stimulating pool in the first dimension. The clone that is present in a positive pool in both dimensions of library is designated the stimulating clone. (TIF) [file ppat.1002989.s002.tif]

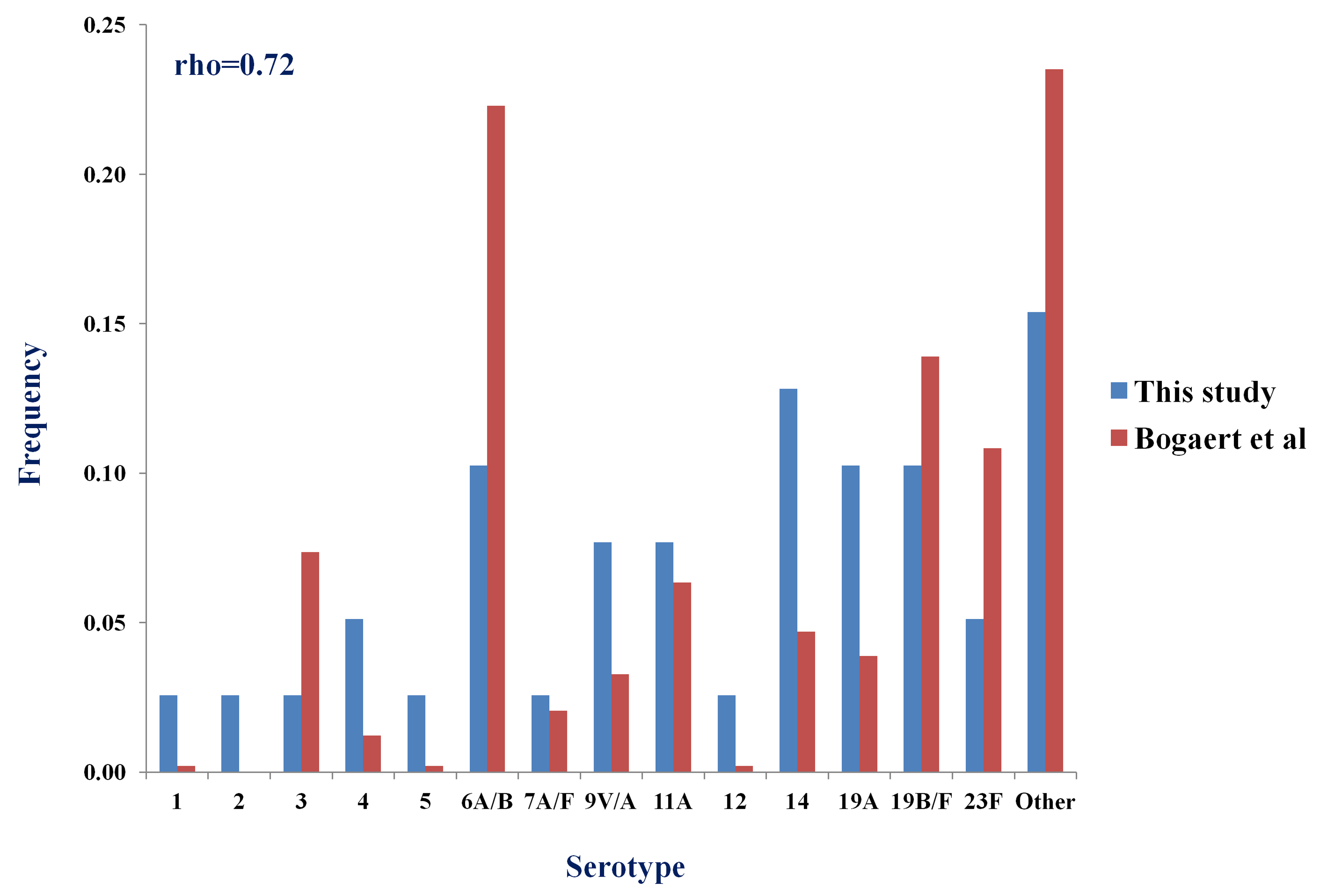

Supplement: Figure S3 — Serotype distribution of strains analyzed in this study is compared with what was reported for human carriage by Bogaert et al [33]. The Spearman's rank correlation coefficient (rho) is shown. (TIF) [file ppat.1002989.s003.tif]
