# Supplementary material for: Distinct Effects on Diversifying Selection by Two Mechanisms of Immunity against Streptococcus pneumoniae
Source: PLoS Pathog. 2012 Nov 8;8(11):e1002989. doi: 10.1371/journal.ppat.1002989 (PMC3493470; doi:10.1371/journal.ppat.1002989)
Supplement: Text S1 — The file includes supplementary methods, supplementary figure legends, table S1: genomic sequence data used in this study, table S2: distribution of codon sites under diversifying selection, table S3: effects of sequence alignment and evolution model on the detection of diversifying selection, and table S4: effects of sequence alignment and evolution model on the GEE analysis. (DOCX) [file ppat.1002989.s004.docx]

**SUPPORTING INFORMATION**

**Supplementary Methods**

**Media and Reagents**

cAIMV contains AIMV media (Invitrogen, Grand Island, NY) supplemented with 5% FBS (Hyclone, Logan, Utah), 1 mM sodium pyruvate, 2 mM L-glutamine, 10 μM MEM non-essential amino acids, 50 μM β-mercaptoethanol, 200 U/ml penicillin, and 200 μg/ml streptomycin. TCM media contains 50% RPMI-1640 media (Invitrogen, Grand Island, NY) and 50% Alpha medium (Invitrogen, Grand Island, NY) supplemented with 10% FBS (Hyclone, Logan, Utah), 10 mM HEPES buffer, 1 mM sodium pyruvate, 2 mM L-glutamine, 10 μM MEM non-essential amino acids, 50 μM β-mercaptoethanol, 200 U/ml penicillin, and 200 μg/ml streptomycin.

**T_H_17 culture enrichment**

Peripheral cell samples were acquired from 58 de-identified subjects in the form of leukopaks or apheresis collection collars from Life Blood Technologies or Children’s Hospital Boston respectively. PBMC were isolated from the sample by Ficoll-Hypaque gradient (Sigma-Aldrich, St. Louis, MO). 2x10^8^ cells were used for T_H_17 enrichment; the rest were frozen for later elicitation of monocyte-derived dendritic cells (MoDC). *S. pneumoniae*-specific T_H_17 cells were enriched by first negatively sorting CD4^+^ T cells using MACS magnetic beads according to the manufacturer’s protocol (Miltenyi Biotec Inc, Auburn, CA). The flow through was used for further T cell enrichment, the bound fraction was used to elicit MoDCs for stimulating the enriched T cells. The CD4^+^ T cells were rested overnight in cAIMV media. The following day the cells were enriched for cells secreting IL-17A using a previously published cytokine capture method ([1](#_ENREF_1)), which typically yielded a 100-fold increase in IL-17A secretion from the enriched cells (Fig. S1A). Briefly, the cells were activated with PMA and ionomycin in cAIMV for 3.5 hours. The cells were then labeled with a bifunctional antibody conjugate consisting of biotinylated anti-IL-17A (eBIoscience, San Diego, CA) and anti-CD45 (Invitrogen, Grand Island, NY) antibodies bridged by avidin (Invitrogen, Grand Island, NY). The cells were then incubated for a further 1.5 hours on a rotator at 37°C. Captured IL-17A was then labeled with a detection IL-17A antibody conjugated to PE (eBIoscience, San Diego, CA). PE-positive cells were sorted using anti-PE MACS beads. 10^5^ cells/well of the enriched cells were added to a 96 well plate containing 10^5^ irradiated feeder cells/well from the same donor in cAIMV containing 100 U/mL rhIL-2.

MoDCs were elicited by enriching for monocytes by adhering cells to a tissue culture treated surface for 1 hour at 37°C, washing the surface and adding TCM media containing 400 IU/mL IL-4 and 300 IU/mL GM-CSF. Half the media was replaced with new cytokine containing media on day 2 and day 4 and the cells were harvested on day 5-6 by washing the surface and harvesting the resuspended cells.

After three days of culture, the enriched cells were added to irradiated MoDCs that had previously been pulsed with inactivated *S. pneumoniae* at an MOI of 100:1 for 1-2 hours. The cells were cultured for 10-14 more days, refreshing IL-2 every two days and splitting the cells to maintain a density of roughly 10^6^ cells/mL. The exposure to inactivated *S. pneumoniae* led to a further increase in the amount of IL-17A secreted when the cells were restimulated with pneumococcus (Fig. S1B). 36 cell lines were successfully established from the 58 initial samples.

**Screening the *S. pneumoniae* expression library**

The construction, maintenance and induction of the *S. pneumoniae* library has been previously published ([2](#_ENREF_2)). The clonal library was pooled with two different methods to create the two-dimensional pooled library. The first dimension was created by combining library clones present in the same well in four consecutive plates of the library when it was arrayed in a 96 well format. The second dimension was created by pooling four consecutive rows on the same plate (Fig. S2). Thus, each library clone was included in two separate pools of four clones each, with only one clone in common between any two pools. In this way, if both pools stimulate a positive T cell response in the screen, the clone responsible was immediately identifiable. 2x10^6^ bacteria from each pool were pulsed onto 2x10^4^ MoDCs adhered to poly-lysine treated 384-well plates. Three wells on each plate were pulsed with inactivated *S. pneumoniae* and three received equivalent amounts of *E. coli* expressing GFP. After an hour incubation at 37°C and fixation of the MoDCs with 1% paraformaldehyde, 5x10^4^ enriched T_H_17 cells were added to each well. The plates were incubated at 37°C for three days. The supernatant was then harvested and analyzed for IL17A content in duplicate using an ELISA kit (eBIoscience, San Diego, CA) according to the manufacturer’s recommendations.

**Supplementary Figure Legends**

**Figure S1.** Enrichment of *S. pneumoniae*-specific T_H_17 cells. (A) CD4^+^ T cells purified from PBMCs by magnetic sorting were further enriched for IL-17A secreting cells through IL-17A capture and sorting. A portion of the enriched cells and unsorted CD4^+^ T cell population were nonspecifically expanded with α-CD3/α-CD28 antibody-coated beads for 12 days in the presence of IL-2 and then activated with PMA/ionomycin in duplicate wells. The average IL-17A concentration in the supernatant was measured by ELISA after three days of incubation and is plotted for each T cell population. (B) A portion of the two T cell populations nonspecifically expanded in part (a) were added to MoDCs that had been pulsed for one hour with inactivated *S. pneumoniae*. After 12 days, both the nonspecifically activated and *S. pneumoniae*-pulsed MoDC-activated T cells were added to fresh MoDCs that had been pulsed for two hours with either *S. pneumoniae* or media alone and then fixed with paraformaldehyde prior to addition of the T cells. The IL-17A concentration in the supernatant after three days of incubation was measured by ELISA and is displayed for each T cell population. US = unsorted, T_H_17 = enriched for T_H_17 cells, NS = nonspecifically activated for expansion, WCV = activated with *S. pneumoniae*-pulsed MoDCs for expansion. Error bars = 1 SD.

**Figure S2.** Pooling strategy for the clonal library. Each set of four consecutive plates in the clonal library were pooled with two different methods to create a two-dimensional library. The first dimension was created by pooling the same well in the four consecutive plates. The second dimension was created by pooling four consecutive rows on the same plate. The individual clone responsible for inducing a T cell response to a pool was identified by examining the four pools in the second dimension that contain one of the clones present in the stimulating pool in the first dimension. The clone that is present in a positive pool in both dimensions of library is designated the stimulating clone.

**Figure S3.** Serotype distribution of strains analyzed in this study is compared with what was reported for human carriage by Bogaert *et al* ([3](#_ENREF_3)). The Spearman's rank correlation coefficient (rho) is shown.

**Table S1 Genomic Sequence data used in this study**

| Accession Number | Strain Name | Serotype |
| --- | --- | --- |
| NC_003028 | TIGR4 | 4 |
| NC_003098 | R6 | NT |
| NC_008533 | D39 | 2 |
| NC_010380 | Hungary19A | 19A |
| NC_010582 | CGSP14 | 14 |
| NC_011072 | G54 | 19F |
| NC_011900 | ATCC_700669 | 23F |
| NC_012466 | JJA | 14 |
| NC_012467 | P1031 | 1 |
| NC_012468 | 70585 | 5 |
| NC_012469 | Taiwan19F | 19F |
| NC_014251 | TCH8431/19A | 19A |
| NC_014494 | AP200 | 11A |
| NC_014498 | 670-6B | 6B |
| NZ_AAGY00000000 | TIGR | 4 |
| NZ_AAZZ00000000 | CGS3BS71 | 3 |
| NZ_ABAA00000000 | CGS6BS73 | 6 |
| NZ_ABAB00000000 | CGS9BS68 | 9 |
| NZ_ABAC00000000 | CGS11BS70 | 11 |
| NZ_ABAD00000000 | CGS14BS69 | 14 |
| NZ_ABAE00000000 | CGS6BS74 | 6 |
| NZ_ABAF00000000 | CGS19BS75 | 19 |
| NZ_ABAG00000000 | CGS23BS72 | 23 |
| NZ_ABFS00000000 | CDC1873-00 | 6A |
| NZ_ABFT00000000 | CDC1087-00 | 7F |
| NZ_ABGE00000000 | SP195 | 9V |
| NZ_ABGF00000000 | CDC0288-04 | 12F |
| NZ_ABGG00000000 | CDC3059-04 | 19A |
| NZ_ABGH00000000 | MLV-016 | 11A |
| NZ_ABWA00000000 | CGS BS458 | 14 |
| NZ_ABWB00000000 | CGS BS457 | NT |
| NZ_ABWC00000000 | CGS BS397 | NT |
| NZ_ABWQ00000000 | BS292 | 14 |
| NZ_ABWU00000000 | BS293 | 9V |
| NZ_ABZC00000000 | CCRI-1974 | NT |
| NZ_ABZT00000000 | CCRI 1974M2 | NT |
| NZ_ACNU00000000 | Canada-MDR-19A | 19A |
| NZ_ACNV00000000 | Canada-MDR-19F | 19F |
| NZ_ADHN00000000 | BS455 | NT |

**Table S2 Distribution of codon sites under diversifying selection**

| Cellular Function | Codon sites^a^ showing dN/dS>1 | | Fold Enrichment |
| --- | --- | --- | --- |
|  | **Yes** | **No** |  |
|  |  |  |  |
| Cell envelope | 262 | 61163 | **2.07** |
| Cellular processes | 60 | 24027 | **1.20** |
| Energy metabolism | 99 | 43215 | **1.10** |
| Biosynthesis of cofactors, prosthetic groups, and carriers | 23 | 11038 | **1.00** |
| Protein synthesis | 60 | 32238 | **0.90** |
| DNA metabolism | 61 | 33844 | **0.87** |
| Hypothetical proteins | 89 | 52680 | **0.81** |
| Amino acid biosynthesis | 23 | 13925 | **0.80** |
| Transport and binding proteins | 117 | 71535 | **0.79** |
| Central intermediary metabolism | 6 | 3728 | **0.78** |
| Regulatory functions | 40 | 26044 | **0.74** |
| Protein fate | 31 | 26773 | **0.56** |
| Transcription | 9 | 9439 | **0.46** |
| Purines, pyrimidines, nucleosides, and nucleotides | 7 | 11306 | **0.30** |
| Fatty acid and phospholipid metabolism | 1 | 5628 | **0.09** |
| Mobile and extrachromosomal element functions | 0 | 798 | **0.00** |
|  |  |  |  |
| Total | 888 | 427381 | **1.00** |

**a** only orthologous groups whose function can be inferred from the TIGR4 genome annotation were included in this analysis (a total of 428269 codon sites in 1461 genes).

**Table S3.** Effects of sequence alignment and evolution model on the detection of diversifying selection

| Analysis |  |  | Alignment algorithm |  |  | Evolution model |  |  | Proportion of gene with signs of diversifying selection | | | Proportion of codon site showing dN/dS>1 | | | |
| --- | --- | --- | --- | --- | --- | --- | --- | --- | --- | --- | --- | --- | --- | --- | --- |
|  |  |  |  |  |  |  |  |  | **Non-antigen (n=1648)** | **T cell antigen (n=48)** | **Antibody antigen (n=80)** | **Non-antigen (n=453618)** | **T cell antigen (n=18065)** | **Antibody antigen (n=42928)** | **Antibody epitope (n=8992)** |
|  | | |  | | |  | | |  |  |  |  |  |  |  |
| 1 | | | PRANK | | | PAML | | | 0.21 | 0.17 | 0.34* | 0.0018 | 0.0033 | 0.0042 | 0.0062 |
|  | | |  | | |  | | |  |  |  |  |  |  |  |
| 2 | | | PRANK | | | Omegamap | | | 0.23 | 0.21 | 0.46*** | 0.0017 | 0.025 | 0.037 | 0.043 |
|  | | |  | | |  | | |  |  |  |  |  |  |  |
| 3 | | | ClustalW | | | PAML | | | 0.30 | 0.33 | 0.42* | 0.0065 | 0.0089 | 0.0096 | 0.013 |
|  | | |  | | |  | | |  |  |  |  |  |  |  |
| 4 | | | ClustalW | | | Omegamap | | | 0.41 | 0.44 | 0.70*** | 0.028 | 0.039 | 0.046 | 0.051 |

* p<0.05; ***p<0.001; compared with non-antigen; Fisher’s Exact test

**Table S4.** Effects of sequence alignment and evolution model on the GEE analysis.

| Analysis |  |  | Alignment algorithm |  |  | Evolution Model |  |  | Generalized Estimating Equation (GEE) model^a^ for the probability that a gene shows signs of being under diversifying selection | | | | |
| --- | --- | --- | --- | --- | --- | --- | --- | --- | --- | --- | --- | --- | --- |
|  |  |  |  |  |  |  |  |  | **Variable** | **Estimate** | **S.E.^b^** | **Odds Ratio** | **p value** |
|  | | |  | | |  | | |  |  |  |  |  |
| 1 | | | **PRANK** | | | **PAML** | | | Intercept | -6.31 | 0.065 | - | - |
|  |  |  |  |  |  |  |  |  | **Antibody** | **0.80** | **0.25** | **2.33** | **0.0016*** |
|  |  |  |  |  |  |  |  |  | **T cell** | **0.45** | **0.33** | **1.57** | **0.17** |
|  | | |  | | |  | | |  |  |  |  |  |
| 2 | | | **PRANK** | | | **OmegaMap** | | | Intercept | -6.37 | 0.070 | - | - |
|  |  |  |  |  |  |  |  |  | **Antibody** | **0.51** | **0.26** | **1.67** | **0.038*** |
|  |  |  |  |  |  |  |  |  | **T cell** | **0.11** | **0.32** | **1.12** | **0.66** |
|  | | |  | | |  | | |  |  |  |  |  |
| 3 | | | **Clustalw** | | | **PAML** | | | Intercept | -5.03 | 0.086 | - | - |
|  |  |  |  |  |  |  |  |  | **Antibody** | **0.37** | **0.23** | **1.45** | **0.11** |
|  |  |  |  |  |  |  |  |  | **T cell** | **0.25** | **0.31** | **1.28** | **0.42** |
|  | | |  | | |  | | |  |  |  |  |  |
| 4 | | | **Clustalw** | | | **OmegaMap** | | | Intercept | -3.55 | 0.083 | - | - |
|  |  |  |  |  |  |  |  |  | **Antibody** | **0.62** | **0.24** | **1.85** | **0.0095*** |
|  |  |  |  |  |  |  |  |  | **T cell** | **0.23** | **0.43** | **1.26** | **0.60** |

a Assuming a distance-weighted correlation structure (arl)

b Standard error is reported for the estimate and not for the odds ratio

**References**

1. Streeck H*, et al.* (2008) Rapid ex vivo isolation and long-term culture of human Th17 cells. *Journal of immunological methods* 333(1-2):115-125.

2. Moffitt KL*, et al.* (2011) T(H)17-based vaccine design for prevention of Streptococcus pneumoniae colonization. *Cell host & microbe* 9(2):158-165.

3. Bogaert D*, et al.* (2001) Pneumococcal carriage in children in The Netherlands: a molecular epidemiological study. *Journal of clinical microbiology* 39(9):3316-3320.
